# Breastfeeding and behavior disorders among children and adolescents: a systematic review

**DOI:** 10.11606/S1518-8787.2018052000439

**Published:** 2018-01-29

**Authors:** Wanêssa Lacerda Poton, Ana Luiza Gonçalves Soares, Elizabete Regina Araújo de Oliveira, Helen Gonçalves

**Affiliations:** I Universidade Vila Velha. Departamento de Medicina. Vila Velha, ES, Brasil; II University of Bristol. Bristol Medical School. Bristol, United Kingdom; III Universidade Federal do Espírito Santo. Programa de Pós-Graduação em Saúde Coletiva. Vitória, ES, Brasil; IV Universidade Federal de Pelotas. Programa de Pós-Graduação em Epidemiologia. Pelotas, RS, Brasil

**Keywords:** Breast Feeding, Mental Disorders, Child Development, Child Behavior, Adolescent Development, Adolescent Behavior, Review

## Abstract

**OBJECTIVE:**

This systematic review study aimed to assess the evidence available for the association between breastfeeding and behavior disorders in childhood and adolescence.

**METHODS:**

The search was carried out in the PubMed, Lilacs, and PsycINFO databases up to December 2016. Inclusion criteria were as follows: prospective, retrospective and cross-sectional studies assessing the association between breastfeeding and behavior disorders in childhood or adolescence, using psychometric tests, carried out in humans and published in Portuguese, English, or Spanish. The search was performed in several stages by two independent researchers using pre-established criteria.

**RESULTS:**

Eighteen studies met the inclusion criteria. Breastfeeding for a period equal to or higher than three or four months seemed to be inversely associated with total behavior and conduct disorders in childhood; however, the association remains unclear for other behavior disorders. Only four studies assessed behavior disorders in adolescence, and when an association was found, it was likely to be positive. The duration of breastfeeding seemed to be more important than the exclusive or non-exclusive pattern of breastfeeding.

**CONCLUSIONS:**

Breastfed children for at least three to four months had fewer total behavior and conduct disorders in childhood. Further studies are needed to better understand this association, particularly in adolescence and involving other behavioral profiles.

## INTRODUCTION

A number of studies have shown the benefits of breastfeeding for both children and mothers, regardless of socioeconomic status^1^ ^–^ ^4^. Breastfeeding reduces the risk of some diseases that may occur at different stages of life^1^ ^–^ ^4^. A recent meta-analysis has shown that breastfeeding not only protects the child against infections but may also reduce the risk of overweight and diabetes and protect the mother against breast and ovarian cancer, and type-2 diabetes^4^. Moreover, children breastfed for at least six months have a higher intelligence quotient (IQ) in childhood^5^ ^–^ ^7^, and this effect is maintained into adolescence^7^ ^,^ ^8^ and adulthood^7^ ^–^ ^9^.

In the quest for more evidence of the advantages of breastfeeding, researchers have increasingly investigated the relationship between breastfeeding and behavior disorders in childhood and adolescence since the 1980s^10^ ^,^ ^11^. Some studies have reported benefits of breastfeeding on emotional and behavioral development in children and adolescents^12^ ^–^ ^16^, whilst others with the same aim have not found any association^17^ ^–^ ^19^.

The term behavior disorders are used in different ways by researchers^20^. The most common use involves deviation of social behavior or norms, which is found in deficits or surplus behavior impairing individual interaction with other children and adults^21^ ^,^ ^22^. Behavior disorders have disruptive characteristics. Conduct disorder, attention deficit and challenging behavior are the most common manifestations of this disorders^23^.

Summarizing the evidence available for the association between breastfeeding and behavior disorders in childhood and adolescence is indeed challenging, due to the inconsistent results found in the literature. A review of these results may indicate whether there is a link between breastfeeding and lower risk of behavior disorders, which in turn may encourage proper breastfeeding. This systematic review aims to discuss the evidence available for an association between duration of breastfeeding and behavior disorders during childhood and adolescence.

## METHODS

### Literature Search

The findings included in this review describe the types of behavior disorders in childhood and adolescence, which may impair the interaction with peers, or family. For the purposes of this study, breastfeeding was considered as the exposure, and studies assessing any breastfeeding duration and pattern (exclusive, predominant, or partial) were included. Studies that classified breastfeeding as predominant or partial were considered non-exclusive breastfeeding in this review.

We carried out a systematic search in PubMed, Lilacs, and PsycINFO databases, including papers published up to December 25, 2016. Literature search was carried out using the following terms for breastfeeding: breastfeed*, breast-feed*, breastfed, breast-fed, bottle feed*, bottle fed, human milk, lactancy, infant feeding, formula milk, infant formula, formula feed, formula fed, weaning. The breastfeeding terms were combined with the following keywords for behavioral outcomes: mental health, mental health problems, mental disorders, mental disorder, mental disability, behavior, behavior, behavioral, behavior disorder, social behavior, health behavior, infant behavior, conduct disorder.

The search strategy and the results obtained are summarized in Figure.

**Figure:**
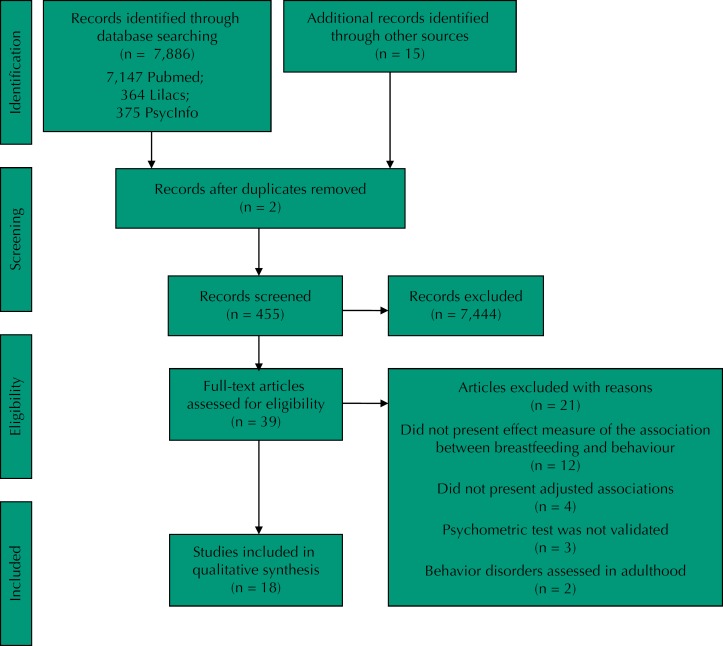
Flowchart of the search strategy used in this systematic review.

### Selection Strategy

All references were imported to a library using EndNote software, and duplicates were excluded before title screening. In order to avoid selection bias, papers were screened by two independent researchers (WLP and ERAO). Agreement between researchers was checked at every stage of the review. In case of disagreement, the papers were reassessed by both researchers to determine whether the study should be included (or not) in the review.

The second stage consisted of a careful reading of all abstracts of the selected titles. After abstract reading, those meeting the inclusion criteria were selected for full-text reading. Additionally, all references to the papers selected were verified.

The three mandatory inclusion criteria for were as follows: 1) having breastfeeding as the exposure (any period or pattern of breastfeeding); 2) having behavior disorders as the outcome; 3) assessing behavior using validated psychometric tests or international classification of behavior problems for children or adolescents (up to 19 years old^24^).

All studies included were conducted on healthy and full-term children. Studies were excluded if they assessed preterm or low-birth-weight infants, if they involved fatty acids or any other type of supplementation, or if the behavior was assessed in adulthood. We also excluded studies that did not present effect measures adjusted for confounders and those assessing behavior by non-validated scales to minimize the inclusion of studies that may have a misclassification of behavior disorders, which could affect the effect measure of the association^25^. Papers published in languages other than English, Portuguese, or Spanish were excluded.

Data extracted from the papers were: year and country of study, population, and design; classification of breastfeeding; type of behavior assessed and instrument used; covariates; and effect measures of the association between breastfeeding and behavior disorders.

### Quality Assessment of the Studies

The methodological quality of the selected papers was assessed using the instrument used by Horta et al.^2^ This seven-question instrument scored/involved/measured: (a) losses to follow-up (0: more than 15% of losses to follow-up; 1: 15% of less of losses to follow-up); (b) type of study (0: observational; 1: randomized); (c) birth cohort (0: no; 1: yes); (d) length of recall of breastfeeding duration (0: ≥ 3 years; 1: < 3 years); (e) source of information on breastfeeding (0: records; 1: interview with subjects; 2: mothers); (f) control for confounding (0: none; 1: socioeconomic or demographic variables; 2: socioeconomic and demographic variables; 3: socioeconomic, demographic, and maternal variables – i.e., mental health, smoking in pregnancy, alcohol intake in pregnancy, stressful events in pregnancy, and others); (g) control for possible mediating variables (0: yes; 1: no). The item “b” was not used in our study, as it would be unethical to randomly allocate some infants to receive breastfeeding and some to be deprived of it. The score could then vary from zero to nine, and the higher the score obtained, the better the methodological quality of the study.

Each study was independently evaluated by two reviewers for each of the quality items, and disagreements were solved by consensus. No study was excluded based on this score alone; however, it was considered in the interpretation of the results.

## RESULTS

### Study Characteristics

The search strategy identified 7,147 references in PubMed, 364 in Lilacs, and 375 in PsycINFO databases, totaling 7,886 titles (Figure). After reading the abstracts, 39 studies met the inclusion criteria for full-text reading. We excluded 21 papers for the following reasons: they did not present effect measure for the association between breastfeeding and behavior (n = 12); they did not present adjusted analysis (n = 4); did not use validated instruments (n = 3); and assessed behavior only in adulthood (n = 2).

Eighteen studies were eligible to be part of this review, and the Table presents the characteristics of the studies assessing the association between breastfeeding and behavior disorders in childhood and adolescence. All studies included in this review were carried out in middle- and high-income countries (Australia^26^ ^–^ ^28^, United Kingdom^29^ ^–^ ^31^, and United States of America^32^ ^–^ ^34^). The majority of the studies had a longitudinal design and started during pregnancy or right after birth: one was an intervention study^35^, one was retrospective^32^, and the others were prospective. Fourteen studies assessed only children^19^ ^,^ ^27^ ^,^ ^29^ ^,^ ^30^ ^,^ ^32^ ^–^ ^41^, three included only adolescents^28^ ^,^ ^31^ ^,^ ^42^, and one assessed both^26^.

**Table:** Papers selected through systematic review on the relation between breastfeeding and behavior disorders

Author, country, year	Study design, sample size, age	Exposition type and duration	Outcome, scale	Confounders	Main results
Taylor^30^ United Kingdom 1984	Cohort 13,135 Child (5 years old)	NEBF Non-breastfed 1–2 months ≥ 3 months	Total behavior (Rutter Behavior Scale)	Sociodemographic: social index (family socioeconomic status, domestic crowding, parental education, tenure of accommodation, type of neighborhood, parental occupation), maternal age, older or younger siblings Mother: smoking in pregnancy, mental health Child: sex, birth weight	1) Total behavior problems (p < 0.05): Non-breastfed: β = 0.01 BF < 1 month: β = 0.04 BF 1–2 months: β = 0.03 BF ≥ 3 months: β = -0.05
Fergusson^37^ New Zealand 1987	Cohort 1,024 Child (6, 7, and 8 years old)	NEBF	Total behavior (Rutter Behavior Scale)	Sociodemographic: family socioeconomic status, family size, maternal marital status, maternal education, maternal ethnicity Child: gestational age, birth weight	1) Maternal ratings: 6 years: β = -0.16, p < 0.001 7 years: β = -0.11, p < 0.001 8 years: β = -0.14, p < 0.001 2) Teacher ratings: 6 years: β = -0.05, p > 0.10 7 years: β = -0.09, p < 0.01 8 years: β = -0.04, p > 0.20 3) Standardized analysis for BF: 6 years: β = -0.07, p > 0.05 7 years: β = -0.14, p < 0.01 8 years: β = -0.05, p > 0.10
Julvez^40^ Spain 2007	Cohort 500 Child (4 years old)	NEBF 2 weeks - < 3 months 3– ≤ 5 months 5–7 months > 7 months *versus* < 2 weeks	Hyperactivity Impulsivity symptoms (ASHD-DSM-IV) Social Competence (California Preschool Social Competence Scale) Abnormal: > 80th percentile	Sociodemographic: parent's social class, parental education level, maternal marital status Mother: maternal parity Child: sex, child's school season e evaluator.	1) Hyperactivity: 2–11.9 weeks: RR = 1.10, 95%CI 0.68–1.78 12–20 weeks: RR = 0.99, 95%CI 0.62–1.58 20.1-28 weeks: RR = 0.68, 95%CI 0.38–1.23 28.1 weeks: RR = 0.48, 95%CI 0.25–0.94 2) Impulsivity symptoms: 2–11.9 weeks: RR = 1.52, 95%CI 0.84–2.73 12–20 weeks: RR = 1.10, 95%CI 0.59–2.04 20.1-28 weeks: RR = 0.79, 95%CI 0.38–1.67 28.1 weeks: RR = 0.70, 95%CI 0.33–1.50 3) Social competence: 2–11.9 weeks: RR = 0.77, 95%CI 0.56–1.05 12–20 weeks: RR = 0.57, 95%CI 0.52–0.66 20.1-28 weeks: RR = 0.16, 95%CI 0.11–0.22 28.1 weeks: RR = 0.44, 95%CI 0.2 7–0.72
Robinson^27^ Australia 2008	Cohort 1,707 Child (5 years old)	NEBF *versus* non-breastfed	Total behavior Internalizing behavior Externalizing behavior (CBCL) Abnormal: > 60th percentile	Sociodemographic: family income, maternal marital status, maternal ethnicity, maternal age, maternal education, number of siblings. Mother: smoking in pregnancy, alcohol in pregnancy, stressful events in pregnancy, baby blues symptoms. Child: gestational age, sex, 5-minute Apgar score.	1) Total behavior problems: 2 years: OR = 0.99, 95%CI 0.95–1.03 5 years: OR = 0.97, 95%CI 0.94–0.99 2–5 years: OR = 1.00, 95%CI 0.96–1.04 2) Internalizing behavior problems: 2 years: OR = 1.02, 95%CI 0.98–1.06 5 years: OR = 0.98, 95%CI 0.96–1.01 2–5 years: OR = 1.04, 95%CI 0.99–1.08 3) Externalizing behavior problems: 2 years: OR = 0.98, 95%CI 0.94–1.01 5 years: OR = 1.00, 95%CI 0.98–1.03 2–5 years: OR = 0.98, 95%CI 0.94–1.02
Kramer^35^ Belarus 2008	Intervention 13,889 Child (6.5 years old)	NEBF	Total behavior Hyperactivity Emotional symptoms Conduct disorder Peer problems Prosocial behavior (SDQ) Abnormal: ≥ 85th percentile	Paired variables: Sociodemographic: maternal age, maternal education, number of siblings Mother: smoking in pregnancy, had breastfed a previous child for at least 3 months, cesarean delivery Child: sex, birth weight, gestational age, 5-minute Apgar score	1) Total behavior problems: parent: β = -0.1; 95%CI -0.7–0.5 teacher: β = -0.5; 95%CI -1.1–0.1 2) Hyperactivity: parent: β = 0.1, 95%CI -0.2–0.3 teacher: β = -0.1, 95%CI -0.4–0.1 3) Emotional symptoms: parent: β = -0.1, 95%CI -0.3–0.1 teacher: β = -0.2, 95%CI -0.3–0.04 4) Conduct disorder: parent: β = 0.0, 95%CI -0.1–0.1 teacher: β = 0.0, 95%CI -0.2–0.1 5) Peer problems: parent: β = 0.0, 95%CI -0.2–0.2 teacher: β = -0.1; 95%CI -0.4–0.1 6) Prosocial behavior: parent: β = 0.1, 95%CI -0.2–0.3 teacher: β = 0.1, 95%CI -0.2–0.5
Kramer^18^ Belarus 2009	Cohort 2,951 Child (6.5 years old)	EBF ≥ 6 months *versus* EBF ≤ 3 months + partial ≥ 6 months	Total behavior Hyperactivity Emotional symptoms Conduct disorder Peer problems Prosocial behavior (SDQ)	Sociodemographic: geographic location, parental education Child: birth weight, age at the follow-up visit, sex	1) Total behavior = problems: parent: β = 0.5, 95%CI -0.05–1.0 teacher: β = 0.0, 95%CI -0.6–0.6 2) Hyperactivity: parent: β = 0.2, 95%CI -0.02–0.4 teacher: β = -0.1, 95%CI -0.4–0.2 3) Emotional symptoms: parent: β = 0.1, 95%CI -0.1 -0.3 teacher: β = 0.0, 95%CI -0.2–0.2 4) Conduct disorder: parent: β = 0.1, 95%CI -0.1–0.2 teacher: β = 0.0, 95%CI -0.2–0.2 5) Peer problems: parent: β = 0.1, 95%CI -0.04–0.3 teacher: β = 0.1, 95%CI -0.1–0.3 6) Prosocial behavior: parent: β = 0.0, 95%CI -0.2–0.2 teacher: β = -0.1, 95%CI -0.3–0.2
Oddy^26^ Australia 2010	Cohort 2,868 Child (2, 5, and 8 years old) Adolescent (10, and 14 years old)	NEBF < 6 months *versus* ≥ 6 months	Total behavior Internalizing behavior Externalizing behavior (CBCL) Abnormal: ≥ 60th percentile	Sociodemographic: family income, maternal marital status, maternal age, maternal education Mother: smoking in pregnancy, stressful events in pregnancy, postnatal depression Child: sex, proportion of optimal birth weight	1) Total behavior problems - 2 to 14 years: NEBF < 6 m: OR = 1.33, 95%CI 1.09–1.62 β = 1.45, 95%CI 0.59–2.30, p = 0.001 2) Internalizing behavior problems - 2 to 1 4 years: NEBF < 6 m: OR = 1.21, 95%CI 1.00–1.46 β = 0.92, 95%CI 0.1 5–1.68, p = 0.019 3) Externalizing behave problems - 2 to 14 years: NEBF < 6 m: OR = 1.23, 95%CI 1.01–1.49 β = 1.33, 95%CI 0.51–2.15, p = 0.001
Chiu^39^ Taiwan 2011	Cohort 14,621 Child (18 months)	NEBF < 1 month 1 - < 3 months 3- < 6 months ≥ 6 months *versus* non-breastfed	Personal/social skills (Denver Development Screening Test) Abnormal: > 90th percentile	Sociodemographic: family structure, income and urbanicity, maternal age, maternal education, maternal country of origin, maternal working situation Mother: smoking in pregnancy, gestational age Child: birth order, birth weight, sex, primary caregiver	1) Personal/social skills: < 1 month: OR = 1.02, 95%CI 0.86–1.20 1 at < 3 months: OR = 0.97, 95%CI 0.82–1.15 3 at < 6 months: OR = 0.83, 95%CI 0.68–1.00 ≥ 6 months: OR = 0.76, 95%CI 0.64–0.90
Heikkila^29^ United Kingdom 2011	Cohort 9,525 term children Child (5 years old)	NEBF < 2 months 2- < 4 months ≥ 4 months EBF < 2 months 2- < 4 months ≥ 4 months *versus* non-breastfed	Total behavior Hyperactivity Emotional symptoms Conduct disorder Peer problems Prosocial behavior (SDQ) Abnormal: > 90th percentile	Sociodemographic: family socioeconomic position, maternal age, maternal education, baby's birth order Mother: mental health, smoking in pregnancy, alcohol in pregnancy Child: mother-baby attachment, admission to neonatal unit, type of childcare the child attended and age when the child started childcare	1) Total behavior problems: NEBF ≥ 4 months: OR = 0.67, 95%CI 0.54–0.83 EBF ≥ 4 months: OR = 0.61, 95%CI 0.45–0.83 2) Hyperactivity: NEBF 2- < 4 months: OR = 0.65, 95%CI 0.43–1.00 EBF 2- < 4 months: OR = 0.68, 95%CI 0.48–0.95 3) Emotional symptoms: NEBF ≥ 4 months: OR = 0.78, 95%CI 0.55–1.10 EBF ≥ 4 months: OR = 0.63, 95%CI 0.39–1.00 4) Conduct disorder: NEBF ≥ 4 months: OR = 0.70, 95%CI 0.56–0.89 EBF ≥ 4 months: OR = 0.70, 95%CI 0.56–0.89 5) Peer problems: NEBF ≥ 4 months: OR = 0.90, 95%CI 0.75–1.08 EBF ≥ 4 months: OR 0.81, 95%CI 0.65–1.02 6) Prosocial behavior: NEBF ≥ 4 months: OR = 0.85, 95%CI 0.45–1.63 EBF ≥ 4 months: OR = 0.48, 95%CI 0.20–1.18
Shelton^32^ United Kingdom and United States 2011	Retrospective Cohort 870 Child (6 years old)	NEBF	Conduct disorder (SDQ)	Sociodemographic: maternal education Mother: smoking in pregnancy, antisocial behavior, multiple birth status Child: time in a special care baby unit, birth weight, age, sex	1) Conduct disorder: total sample: β = -0.28, 95%CI -0.49- -0.08 genetically related mother-child: β = -0.33, 95%CI -0.56- -0.09 genetically unrelated mother-child: β = -0.09, 95%CI -0.55–0.38
Cable^31^ United Kingdom 2012	Cohort 6,205 Adolescent (10 years old)	NEBF ≥ 1 month *versus* < 1 month	Total behavior (Rutter Behavior Scale)	Sociodemographic: maternal marital status, parity, maternal age, maternal education, baby's birth order	1) Overall effect of NEBF: men: β = -0.025, p > 0.05 women: β = -0.203, p < 0.05
Hayatbakhsh^28^ Australia 2012	Cohort 4,502 Adolescent (14 years)	NEBF < 4 months ≥ 4 months *versus* non-breastfed	Anxiety/depression Withdrawal problems Social problems Somatic complaints Thought problems Attention problems Aggression Delinquency (YSR)	Sociodemographic: maternal age, maternal education, maternal marital status, planned pregnancy Mother: anxiety, depression, smoking in pregnancy, alcohol in pregnancy Child: sex	1) Anxiety/depression: NEBF < 4 months: β = -0.14, 95%CI -0.49–0.20 NEBF ≥ 4 months: β = -0.35, 95%CI -0.69–0.01 2) Social problems: NEBF < 4 months: β = -0.1 7, 95%CI -0.34- -0.01 NEBF ≥ 4 months: β = -0.26, 95%CI -0.43- -0.09 3) Attention problems: NEBF < 4 months: β = -0.23, 95%CI -0.47–0.02 NEBF ≥ 4 months: β = -0.39, 95%CI -0.64- -0.14 4) Aggression: NEBF < 4 months: β =-0.15, 95%CI -0.59–0.29 NEBF ≥ 4 months: β = -0.48, 95%CI -0.93- -0.04 5) Delinquency: NEBF < 4 months: β = -0.08, 95%CI -0.27–0.12 NEBF ≥ 4 months: β = -0.16, 95%CI -0.36–0.04 Did not present adjusted analyses for: withdrawal problems, somatic complaints, thought problems.
Kwok^42^ Hong Kong 2013	Cohort 5,598 Adolescent (11 years old)	NEBF < 3 months EBF ≥ 3 months *versus* non-breastfed	Total behavior Conduct disorder Emotional symptoms Hyperactivity (Rutter Behavior Scale) Abnormal: ≥ 97th percentile	Sociodemographic: family income, mother's birthplace, parental age, parental education, baby's birth order Child: sex, birth weight-for-gestational age, age at assessment, secondhand smoke exposure.	1) Total behavior problems: NEBF < 3 months: OR = 1.25, 95%CI 1.10–1.44, β = 0.10, 95%CI 0.05–0.16 EBF ≥ 3 months: OR = 1.60, 95%CI 0.80–1.40, β = 0.04, 95 % CI -0.07–0.1 5 2) Conduct disorder: NEBF < 3 months: OR = 1.63, 95%CI 1.20–2.20, β = 0.10, 95 %CI 0.04–0.16 EBF ≥ 3 months: OR = 1.30, 95%CI 0.69–2.46, β = 0.13, 95 %CI 0.02–0.25 3) Emotional symptoms: NEBF < 3 months: OR = 1.05, 95%CI 0.82–1.35, β = 0.04, 95%CI -0.02–0.10 EBF ≥ 3 months: OR = 0.94, 95%CI 0.55–1.62, β = -0.01, 95%CI -0.12–0.10 4) Hyperactivity: NEBF < 3 months: OR = 1.26, 95%CI 0.85–1.89, β = 0.06, 95%CI 0.01–0.12 EBF ≥ 3 months: OR = 0.68, 95%CI 0.24–1.92, β = -0.07, 95%CI -0.19–0.04
McCrory^38^ Ireland 2013	Cohort 11,134 Child (9 months old)	EVER BF NEBF ≤ 1 week 2 weeks at < 1 month 1 - < 3 months 3- < 6 months ≥ 6 months EBF ≤ 1 week 2 weeks at < 1 month 1 - < 3 months 3- < 6 months = 6 months > 6 months *versus* non-breastfed	Personal-social ability (ASQ)	Sociodemographic: household social class, occupational classification, maternal marital status, maternal age, maternal educational, ethnicity/racial Mother: smoking in pregnancy Child: birth weight, gestational age	1) Breastfeeding: Ever BF: OR = 1.38, 95%CI 1.23–1.54 NEBF ≤ 1 week: OR = 1.24, 95%CI 1.04–1.48 NEBF 2 weeks at < 1 m: OR = 1.58, 95%CI 1.22–2.05 NEBF 1 at < 3 months: OR = 1.29, 95%CI 1.09–1.53 NEBF 3 at < 6 months: OR = 1.28, 95%CI 1.07–1.54 NEBF ≥ 6 months: OR = 1.64, 95%CI 1.35–2.01 2) Exclusive breastfeeding: EBF ≤ 1 week: OR = 0.71, 95%CI 0.52–0.97 EBF < 1 month: OR = 0.91, 95%CI 0.64–1.39 EBF 1 at < 3 months: OR = 0.78, 95%CI 0.58–1.04 EBF 3 at < 6 months: OR = 0.89, 95%CI 0.67–1.18 EBF = 6 months: OR = 1.75, 95%CI 1.35–2.28 EBF > 6 months: OR = 1.27, 95%CI 0.90–1.80
Lind^33^ United States 2014	Cohort 1,442 Child (6 years old)	NEBF < 6 months; NEBF ≥ 6 months + EBF < 3 months; NEBF ≥ 6 months + EBF ≥ 3 months; *versus* non-breastfed	Total behavior Hyperactivity Emotional symptoms Conduct disorder Peer problems Prosocial behavior (SDQ) Abnormal: > 90th percentile	Sociodemographic: poverty-to-income ratio, maternal ethnicity, maternal marital status, maternal age, maternal education, baby's birth order Mother: pre-pregnancy BMI, postpartum depression, smoking in the first year Child: gestational age, participation in the Special Supplemental Nutrition Program, birth weight, sex	1) Total behavior problems: NEBF < 6 m: OR= 1.25, 95%CI 0.67–2.34 NEBF ≥ 6 m + EBF < 3 m: OR = 1.19, 95%CI 0.60–2.35 NEBF ≥ 6 m + EBF ≥ 3 m: OR = 0.76, 95%CI 0.33–1.78 2) Hyperactivity: NEBF < 6 m: OR = 1.45, 95%CI 0.70–3.02 NEBF ≥ 6 m + EBF < 3 m: OR = 1.1 7, 95%CI 0.53–2.54 NEBF ≥ 6 m + EBF ≥ 3 m: OR = 0.99, 95%CI 0.41–2.38 3) Emotional symptoms: NEBF < 6 m: OR = 1.04; 95%CI 0.59–1.81 NEBF ≥ 6 m + EBF < 3 m: OR = 1.39, 95%CI 0.77–2.49 NEBF ≥ 6 m + EBF ≥ 3 m: OR = 0.78, 95%CI 0.39–1.55 4) Conduct disorder: - NEBF < 6 m: OR = 1.08, 95%CI 0.60–1.95 NEBF ≥ 6 m + EBF < 3 m: OR = 0.87, 95%CI 0.45–1.69 NEBF ≥ 6 m + EBF ≥ 3 m: OR = 0.42, 95%CI 0.1 7–1.02 5) Peer problems: NEBF < 6 m: OR = 0.72, 95%CI 0.38–1.34 NEBF ≥ 6 m + EBF < 3 m: OR = 0.50, 95%CI 0.25–1.03 NEBF ≥ 6 m + EBF ≥ 3 m: OR = 0.70, 95%CI 0.33–1.51 6) Prosocial behavior: NEBF < 6 m: OR = 0.65, 95%CI 0.35–1.21 NEBF ≥ 6 m + EBF < 3 m: OR = 0.53, 95%CI 0.27–1.04 NEBF ≥ 6 m + EBF ≥ 3 m: OR = 0.61, 95%CI 0.30–1.27
Liu^36^ China 2014	Cohort 1,267 Child (6 years old)	EBF *versus* NEBF	Emotionally reactive Anxious/Depressed Somatic Complaints Withdrawn Internalizing behavior CBCL	Sociodemographic: house size, neighborhood, parental education, parents occupation, maternal marital status, presence of biological mother Parents: health status, obstetric complication, (bleeding, hypertension, diabetes, caesarian section, difficult birth) Child: birth weight, difficulty breathing	1) Emotionally reactive: EBF: β = -0.092, p = 0.168 2) Anxious/Depressed: EBF: β =-0.091, p = 0.175 3) Somatic complaints: EBF: β = -0.153, p = 0,025 4) Withdrawn: EBF: β = -0.105, p = 0.11 5) Internalizing behavior: EBF: β = -0.137, p = 0.04
Boucher^41^ Spain 2016	Cohort 1,346 Child (4 years old)	NEBF EBF	Attention-Deficit Hyperactivity Disorder DSM-IV	Sociodemographic: social class, parental education, country of mother's birth, Mother: parity, age, smoking in pregnancy, verbal IQ proxy, psychopathology Child: age, sex, cohort, birth weight, child care	1) Attention symptoms: NEBF: β = -0.01, 95%CI -0.02–0.00 EBF: β = -0.01, 95%CI -0.04–0.02 2) Hyperactivity symptoms: NEBF: β = -0.01, 95%CI -0.02–0.00 EBF: β = -0.01, 95%CI -0.04–0.02
Jackson^34^ United States 2016	Cohort 648 pair Child (4 years old)	NEBF NEBF *versus* genetic risk NEBF < 6 months NEBF < 6 months *versus* genetic risk	Conduct disorder Preschool and Kindergarten Behavior Scales (PKBS-2)	Sociodemographic: index annual household income, maternal education, female-headed household, parental involvement Mother: age, postpartum depression Child: age, race, sex, attachment security, birth weight	1) Conduct disorder: NEBF: β = 0.030, p > 0.05 NEBF x genetic risk: β = 0.076, p < 0.01 NEBF < 6 months: β = 0.025, p > 0.05 NEBF < 6 months x genetic risk: β = 0.076, p < 0.01

EBF: exclusive breastfeeding; NEBF: non-exclusive breastfeeding; CBCL: Child Behavior Checklist; SDQ: Strengths and Difficulties Questionnaire; BMI: body mass index; β: linear regression coefficient; ASQ: Ages and Stages Questionnaire; PKBS-2: Preschool and Kindergarten Behavior Scales; YSR: Youth Self Report; β: linear regression coefficient; Df: difference in means; RR: relative risk

The results of the studies are described by life stage – childhood and adolescence – and within each group, according to the pattern and duration of breastfeeding: (i) exclusive breastfeeding (EBF); (ii) non-exclusive breastfeeding < 6 months (NEBF<6mo); (iii) non-exclusive breastfeeding ≥ 6 months (NEBF≥6mo).

### Breastfeeding and Behavior Disorders in Childhood

Fifteen studies assessed the association between breastfeeding and children's behavior^19^ ^,^ ^26^ ^,^ ^27^ ^,^ ^29^ ^,^ ^30^ ^,^ ^32^ ^–^ ^41^, and ten of them found some association between breastfeeding and child behavior^26^ ^,^ ^27^ ^,^ ^29^ ^,^ ^30^ ^,^ ^32^ ^,^ ^34^ ^,^ ^36^ ^,^ ^38^ ^–^ ^40^. Regarding breastfeeding assessment, seven studies assessed EBF^19^ ^,^ ^29^ ^,^ ^33^ ^,^ ^34^ ^,^ ^36^ ^,^ ^38^ ^,^ ^41^ and twelve^26^ ^,^ ^27^ ^,^ ^30^ ^,^ ^32^ ^–^ ^35^ ^,^ ^37^ ^–^ ^41^ studies assessed NEBF.

Nine different instruments were used to assess behavior disorders, and most of the studies used the Strengths and Difficulties Questionnaire (SDQ)^19^ ^,^ ^29^ ^,^ ^32^ ^,^ ^33^ ^,^ ^35^, three used the Child Behavior Checklist (CBCL)^26^ ^,^ ^27^ ^,^ ^36^, and two used the Rutter Behavior Scale^30^ ^,^ ^37^.

#### Exclusive Breastfeeding (EBF)

Different durations of EBF, children's ages and behavior problems were assessed by the researchers. Of the six studies selected, one was carried out in children aged nine months^38^ and five involved children aged 4–6 years^19^ ^,^ ^29^ ^,^ ^33^ ^,^ ^36^ ^,^ ^41^. Three^29^ ^,^ ^36^ ^,^ ^38^ out of six^19^ ^,^ ^29^ ^,^ ^33^ ^,^ ^36^ ^,^ ^38^ ^,^ ^41^ studies investigating EBF found some benefits of breastfeeding on behavior disorders in childhood (somatic complaints, internalized behavior, personal-social ability, total behavior, conduct disorder, hyperactivity, attention symptoms).

Some of the studies assessing EBF for any duration investigated somatic complaints^36^, internalized behavior problems^36^ and Attention-Deficit Hyperactivity Disorder (ADHD)^41^. An inverse association of EBF for any duration with fewer somatic complaints and internalized behavior problems^36^ was found when EBF children were compared to NEBF children, but no association was found for attention-deficit symptoms and hyperactivity^41^.

One study found an inverse association between EBF for two to four months and hyperactivity (OR = 0.68, 95%CI 0.48–0.95), but no association was found for total behavior problems, emotional symptoms, conduct disorder, peer problems, and prosocial behavior^29^. However, when analyzing EBF for ≥ 4 months there was an inverse association with total behavior (OR = 0.67, 95%CI 0.54–0.83) and conduct disorder (OR = 0.70, 95%CI 0.56–0.89)^29^.

Nearly all studies assessing EBF for ≥ 3 months^33^ and EBF for ≥ 6 months^19^ did not find any association with total behavior problems, hyperactivity, emotional symptoms, conduct disorder, prosocial behavior, and peer problems^19^ ^,^ ^33^. Only one study found that children EBF for ≥ 6 months had greater interaction with people and self-care in childhood (higher odds of personal-social ability) when compared to non-breastfed children^38^.

Therefore, there was a great heterogeneity in the studies regarding behavior disorders, which makes it difficult to conclude whether EBF is indeed associated with behavior in childhood. Regarding the methodological quality of the studies, all presented scores ranging from seven to eight, and lower scores were especially due to follow-up losses higher than 15%^29^ ^,^ ^33^ ^,^ ^36^ ^,^ ^38^ and breastfeeding recall greater than three years^36^. Most of the studies that did not find any associations had a small sample size (< 1,500), except for the research undertaken by Kramer^35^.

#### Non-Exclusive Breastfeeding for < 6 months (NEBF<6mo)

All the studies were carried out in children aged between four and eight years old^27^ ^,^ ^29^ ^,^ ^30^ ^,^ ^32^ ^–^ ^35^ ^,^ ^37^ ^,^ ^41^. Nine studies assessed NEBF<6mo^27^ ^,^ ^29^ ^,^ ^30^ ^,^ ^32^ ^–^ ^35^ ^,^ ^37^ ^,^ ^41^ and five of them found an inverse association between NEBF and some behavior domains (total behavior problems, hyperactivity, or conduct disorder)^27^ ^,^ ^29^ ^,^ ^30^ ^,^ ^32^ ^,^ ^34^.

The association between NEBF<6mo and total behavior disorders was investigated in six studies^27^ ^,^ ^29^ ^,^ ^30^ ^,^ ^33^ ^,^ ^35^ ^,^ ^37^, and three of them showed an inverse association. Children's NEBF for any duration (OR = 0.97, 95%CI 0.94–0.99)^27^, for ≥ 3 months (β = -0.05, p < 0.05)^30^, and for ≥ 4 months (OR = 0.67, 95%CI 0.54–0.83)^29^ had lower odds or lower score of total behavior disorders at five years old when compared to non-breastfed children. Nevertheless, no association was found in two studies investigating NEBF for any duration^35^ ^,^ ^37^ and in one study assessing NEBF<6mo^33^.

Only one study assessed the association between NEBF up to < 4 months and hyperactivity. The results showed that children breastfed between two to 3.9 months had lower odds of hyperactivity (OR = 0.65, 95%CI 0.43–1.00) when compared to those not breastfed^29^. Tree other studies found no association of NEBF for any duration^35^, < 5 months^33^ and < 6 months^40^ with hyperactivity.

Five studies investigated conduct disorder; however, they all used different durations of breastfeeding. Two found lower odds or score of conduct disorder in children who had NEBF for ≥ 4 months (OR = 0.77, 95%CI 0.64–0.93)^29^ and NEBF for any duration (β = -0.28, 95%CI -0.49– -0.08)^32^. However, other studies^33^ ^–^ ^35^, found no evidence for the association between NEBF for < 6 months and conduct disorder in childhood. On the other hand, despite the lack of direct effect of NEBF<6mo on conduct disorder, Jackson found a positive interaction between NEBF<6mo and the genetic risk score for childhood behavioral problems on the risk of conduct disorder (β = 0.076, p < 0.01)^34^.

In summary, there was a wide range of categories of breastfeeding assessed and several types of instrument used to assess behavior. NEBF for at least three or four months had an inverse association with total behavior disorders and conduct disorder, and, although some studies did not find any association, the effect measure pointed towards an inverse relationship. Of the four studies failing to find any association, three of them had a small sample size (< 1,500). The methodological quality scores ranged from five to nine, and the lower points were mostly due to high rates of losses to follow-up^33^ ^,^ ^35^ ^,^ ^37^, long periods of breastfeeding recall^30^ ^,^ ^32^ and lack of control for maternal variables^37^.

#### Non-Exclusive Breastfeeding for ≥ 6 months (NEBF≥6mo)

Five studies^26^ ^,^ ^33^ ^,^ ^38^ ^–^ ^40^ assessed NEBF≥6mo, and four of them^26^ ^,^ ^38^ ^–^ ^40^ found an association with several behavior disorders (total behavior problems, hyperactivity, personal/social ability, social competence, internalizing behavior, and externalizing behavior). Tree studies assessed children aged between two and eight years old^26^ ^,^ ^33^ ^,^ ^40^, and two studies assessed children below two years old^38^ ^,^ ^39^.

One study carried out with children aged two and eight years old found that those who were NEBF<6mo had higher scores for total behavior problems (β = 1.45, p = 0.001), internalizing (β = 0.92, p = 0.019) and externalizing (β = 1.33, p = 0.001) behavior problems than those with NEBF≥6mo^26^. On the other hand, another study assessed six years old children and did not find any difference in the total behavior score associated with breastfeeding^33^.

Children with NEBF > 7 months had a lower risk of hyperactivity at four years old (RR = 0.48, 95%CI 0.25–0.94) than those with NEBF < 2 weeks^40^. However, another study found no association between NEBF≥6mo and hyperactivity when assessing children aged six years old^33^.

Regarding personal/social ability, from the two studies that investigated this behavior component, one found an inverse association between NEBF≥6mo and risk of personal/social developmental delay in children aged 18 months (OR = 0.76, 95%CI 0.64–0.90)^39^, while another found higher odds of personal/social ability in children aged nine months old (OR = 1.64, 95%CI 1.35–2.01)^38^.

One study investigated the association between NEBF for > 7 months and social competence and found that breastfeeding was associated with higher social competence scores (RR = 0.44, 95%CI 0.27–0.72) at four years old^40^.

The results show that NEBF≥6mo had a positive impact on the development of personal/social abilities. There is not clear evidence for the other types of behaviors, such as total behavior and hyperactivity, due to the small number of studies assessing these disorders. A high heterogeneity was observed in the instruments used to assess behavior; yet, all studies scored the same (8 points) in the methodological quality evaluation, and most of the studies had follow-up losses higher than 15%^26^ ^,^ ^33^ ^,^ ^38^ ^,^ ^39^.

### Breastfeeding and Behavior Disorders in Adolescence

Four studies assessed the association between breastfeeding and behavior disorders in adolescents from 10 to 14 years old^26^ ^,^ ^28^ ^,^ ^31^ ^,^ ^42^. Tree of them found an inverse association between breastfeeding and behavior disorders^26^ ^,^ ^28^ ^,^ ^31^, while one study identified breastfeeding as a risk factor for three types of behavior disorders in adolescence (hyperactivity, total behavior disorders, and conduct disorder)^42^.

Regarding breastfeeding characteristics, only one study investigated EBF^42^. The duration of breastfeeding, regardless of whether it was exclusive or not, was set at < 4 months for most of the studies^28^ ^,^ ^31^ ^,^ ^42^.

Two studies used the Rutter Behavior Scale^31^ ^,^ ^42^ while the other studies used the Youth Self Report (YSR)^28^ and CBCL^26^ scales to assess behavior disorders.

#### Exclusive Breastfeeding (EBF)

Only one study investigated EBF ≥ 3 months and found an association with a higher score of conduct disorder at 11 years old (β = 0.13, 95%CI 0.02–0.25)^42^. The same study did not find any association between EBF ≥ 3 months and total behavior problems, emotional symptoms or hyperactivity^42^.

Due to the limited number of studies investigating EBF, it is not possible to conclude whether EBF provides any significant benefits for behavior problems. Regarding the methodological quality characteristics assessed, our study did not adjust the analysis for maternal characteristics, such as maternal mental health.

#### Non-Exclusive Breastfeeding for < 6 months (NEBF<6mo)

Of the three studies that investigated NEBF<6mo^28^ ^,^ ^31^ ^,^ ^42^, two of them found a negative association with behavior disorders in four domains (total behavior, social problems, attention problems, and aggression)^28^ ^,^ ^31^, and one found a positive association with total behavior disorders, hyperactivity and conduct disorders^42^.

A cohort study assessed NEBF ≥ 1 month, and showed that female adolescents who had NEBF ≥ 1 month had lower scores of total behavior problems at 10 years old (β = -0.203, p < 0.05) than those with NEBF for < 1 month^31^, but no association was found for boys^31^. However, another study found a higher odds of total behavior disorders in adolescents (11 years old) who had NEBF < 3 months (OR = 1.25, 95%CI 1.10–1.44)^42^, a higher score of hyperactivity (β = 0.06, 95%CI 0.01–0.12), and a higher odds of conduct disorder (OR = 1.63, 95%CI 1.20–2.20)^42^ when compared to those who were not breastfed.

One study found a negative association between NEBF ≥ 4 months and social problems (β = -0.26, 95%CI -0.43– -0.09), attention problems (β = -0.39, 95%CI -0.64– -0.14) and aggression behavior (β = -0.48, 95%CI -0.93– -0.04)^28^ at 14 years old. That was the only study that assessed these behaviors.

Few studies investigated the association between breastfeeding and behavior disorders in adolescence, which makes it difficult to conclude whether there is a positive impact of breastfeeding duration on these disorders. Moreover, there was a considerable variety of the instruments used to assess behavior. Regarding methodological quality, losses to follow-up higher than 15% were found in all studies^28^ ^,^ ^31^ ^,^ ^42^, and some did not adjust the analysis for socioeconomic^31^ and maternal variables^31^ ^,^ ^42^.

#### Non-Exclusive Breastfeeding for ≥ 6 months (NEBF≥6mo)

Only one study assessed NEBF≥6mo and found that adolescents with NEBF<6mo had higher odds of total behavior disorder (OR = 1.33, 95%CI 1.09–1.62), internalized (OR = 1.21, 95%CI 1.00–1.46), and externalized (OR = 1.23, 95%CI 1.01–1.49) behavior problems than those NEBF≥6mo^26^. Those benefits were observed at both 10 and 14 years old^26^.

Since only a single study assessed duration of breastfeeding equal to or longer than six months, it is not possible to reach any conclusion about its association with behavior disorders in adolescence.

## DISCUSSION

This is the first systematic review that describes the evidence available for the association between breastfeeding (EBF, NEBF<6mo, and NEBF≥6mo) and behavior disorders in childhood and adolescence. The results seem to indicate that breastfeeding for more than three or four months is inversely associated with total behavior and conduct disorder in childhood; however, the findings were not consistent among the studies, and the magnitude of the effect was relatively small. For other types of behaviors, such as hyperactivity, personal/social skills, social competence, somatic complaints, and internalized and externalized behavior problems, this association remains unclear, due to the reduced number of studies assessing these behaviors. Breastfeeding was not associated with some types of behavior in childhood, such as showing negative emotional symptoms, withdrawnness, impulsivity, being anxious/depressed and emotionally reactive, having peer problems, and displaying prosocial behavior. The results suggest that duration of breastfeeding (particularly when longer than three or four months) is more important than the breastfeeding pattern (EBF or NEBF) in the association with child behavior.

Few studies investigated the association between EBF, NEBF<6mo, and NEBF≥6mo and behavior disorders in adolescence, which limited the conclusions for this age group. However, it seems that breastfeeding is associated with lower risk of total behavior disorders and conduct disorders in this age group.

Regarding methodological quality, most of the studies scored equal to or more than six. The differential losses to follow-up among the groups and the long recall period of breastfeeding were the main limitations of the studies. The small differences between the breastfeeding categories and small sample size were also important limitations of the studies, which found no association between breastfeeding and behavior disorders. However, the effect measure of most of the small studies that did not find any association was in the direction of a negative association^33^ ^,^ ^37^ ^,^ ^40^ ^,^ ^41^; thus, the small sample size may have limited the power to identify modest differences. Furthermore, the lack of adjustment for maternal variables, such as maternal mental health, was also a limitation in many studies. These variables are important because postnatal depression, for instance, is associated with both never/short-term breastfeeding and poorer offspring mental health^43^ ^–^ ^45^.

Most of the studies included in this review were birth cohorts and only one was an interventional study^35^. Although randomized clinical trials are considered gold-standard studies for causal inference, since they are less susceptible to selection and information biases^25^, it would not be ethical to randomly assign children to receive maternal or artificial milk. Thus, the randomized assay was designed to allow an intervention strategy in which both groups were exposed to breastfeeding. Although the intervention group had a greater proportion of breastfeeding (49.8%) than the control group (36.1%), the authors reported that a large sample of children would enable them to detect slight differences among the groups^35^. Nonetheless, the results regarding behavior disorders were similar between the intervention and control groups^35^.

Half of the studies assessing total behavior disorders and conduct disorder in children found an inverse association with breastfeeding, especially for duration ≥ three or four months. Some hypotheses have been put forward to account for the inverse relationship between breastfeeding and behavior disorders in childhood. One is the composition of maternal milk, which is rich in key components for child's neurological^46^ ^,^ ^47^, mental, and psychomotor development^48^, and may positively contribute to reducing behavior disorders. Another hypothesis is related to the physical and emotional proximity between mother and child during breastfeeding^49^ ^–^ ^51^, which induces cortisol secretion – a hormone that acts on the response to stress, anxiety, and depression – and, consequently, may affect a child's sociability^52^ ^,^ ^53^. A longer breastfeeding duration may be related to the bond created between the mother and the child, and consequently, may promote benefits in child behavior.

Nevertheless, behavior disorders have multiple causes^54^ and are also strongly related to parental interaction, family environment, and, particularly, the mother's and the child's health and emotional status in the post-natal period, such as postpartum depression, family environment, and child development^28^. For instance, a mother's antisocial behavior has been reported as an important risk factor for the development of conduct disorder in childhood^32^.

Some limitations in this review should be pointed out. Most of the studies used a dichotomized classification of breastfeeding and did not assess a dose-response effect in the association between breastfeeding and behavior disorders. The heterogeneity in breastfeeding patterns (exclusive, predominant, or partial), as well as in the length of recall of breastfeeding, also represents a limitation in some studies. According to Huttly et al.^55^, mothers who breastfeed for up to four weeks and are inquired some time after the interview tend to report having never breastfed. Such recall bias may lead to error in classifying breastfeeding categories and thus underestimate the association between breastfeeding and behavior problems.

Regarding the assessment of behavior disorders, although only studies using psychometric instruments were included in this review, it was difficult to compare some of them, as different instruments were used to assess different behavior profiles. Although SDQ and CBCL have similar psychometric properties, and as such, they facilitate comparisons between each other^56^, other instruments may not be easily comparable. Thus, to minimize this issue, three studies^57^ ^–^ ^59^ that did not use validated tools to assess behavior were excluded. These studies used open questions asked for parents, i.e., “Can your child express emotions appropriately?”, “Can your child get along with others in a group setting?”^57^, along with information from the children's medical records^58^, or observing the children's behavior during the research interview^59^. If these studies were included, the heterogeneity regarding the behavior assessed would be even higher, making it more difficult to arrive at a coherent conclusion. It should be stressed that when a behavior assessment instrument is used, mothers may incur in classification error since those who breastfeed could have a more positive (less critical) view of the child^37^, and this could lead to an inverse association between breastfeeding and behavior disorders. The cut-off point used in the studies to classify behavior disorders also differed among the studies. Some used the threshold suggested by the instrument, whilst others determined their own cut-off point^29^ ^,^ ^33^ ^,^ ^35^ ^,^ ^39^ ^–^ ^40^ ^,^ ^42^, which may have resulted in classification error and thus hindered the comparison of the results.

Given the evidence found for the association between breastfeeding and behavior disorders, our findings suggest that children who are breastfed for at least three or four months may have better total behavior and conduct disorders during childhood, while a longer duration of breastfeeding seems to be more important than the pattern of breastfeeding. The association between breastfeeding and other behaviors, such as hyperactivity, internalized and externalized behavior disorders, among others, should be further investigated. Few studies assessed the association between breastfeeding and behavior disorders in adolescence, and they seem to show a lower risk of total behavior disorders; however, more investigations are needed for a fuller understanding of this issue.

Further studies exploring the association between breastfeeding and behavior disorders should be undertaken, particularly research assessing other types of behavior disorders and in adolescence. Studies carried out in low- and middle-income should be encouraged as well, as different sociodemographic factors may play a role in the relationship between breastfeeding and behavior disorders.
